# Structure and Dynamics of Zika Virus Protease and Its Insights into Inhibitor Design

**DOI:** 10.3390/biomedicines9081044

**Published:** 2021-08-19

**Authors:** Qingxin Li, Congbao Kang

**Affiliations:** 1 Guangdong Provincial Engineering Laboratory of Biomass High Value Utilization, Institute of Biological and Medical Engineering, Guangdong Academy of Sciences, Guangzhou 510316, China; 2 Experimental Drug Development Centre, A*STAR, 11 Biopolis Road, #5-01, Singapore 138670, Singapore

**Keywords:** Zika virus, protease structure, protein dynamics, allosteric inhibitors, structural biology

## Abstract

Zika virus (ZIKV)—a member of the *Flaviviridae* family—is an important human pathogen. Its genome encodes a polyprotein that can be further processed into structural and non-structural proteins. ZIKV protease is an important target for antiviral development due to its role in cleaving the polyprotein to release functional viral proteins. The viral protease is a two-component protein complex formed by NS2B and NS3. Structural studies using different approaches demonstrate that conformational changes exist in the protease. The structures and dynamics of this protease in the absence and presence of inhibitors were explored to provide insights into the inhibitor design. The dynamic nature of residues binding to the enzyme cleavage site might be important for the function of the protease. Due to the charges at the protease cleavage site, it is challenging to develop small-molecule compounds acting as substrate competitors. Developing small-molecule compounds to inhibit protease activity through an allosteric mechanism is a feasible strategy because conformational changes are observed in the protease. Herein, structures and dynamics of ZIKV protease are summarized. The conformational changes of ZIKV protease and other proteases in the same family are discussed. The progress in developing allosteric inhibitors is also described. Understanding the structures and dynamics of the proteases are important for designing potent inhibitors.

## 1. Introduction

Zika virus (ZIKV) belongs to *Flaviviridae,* which contains other important human pathogens, such as dengue, West Nile, yellow fever, and Japanese encephalitis viruses. ZIKV was first isolated in 1947; the viral infection usually causes mild symptoms, which might not require medical treatment [1]. ZIKV infection received attention in recent years, as it was found to transmit from human-to-human and it could result in serious diseases, such as microcephaly in newborns [2] and Guillain–Barré syndrome in adults [3,4]. An outbreak occurred during 2015–2016 and over 2 million people were affected [4,5,6,7]. Effort has been made to develop antivirals and vaccines to combat the virus while no specific medical treatment is available [8,9,10,11].

**Figure 1:**
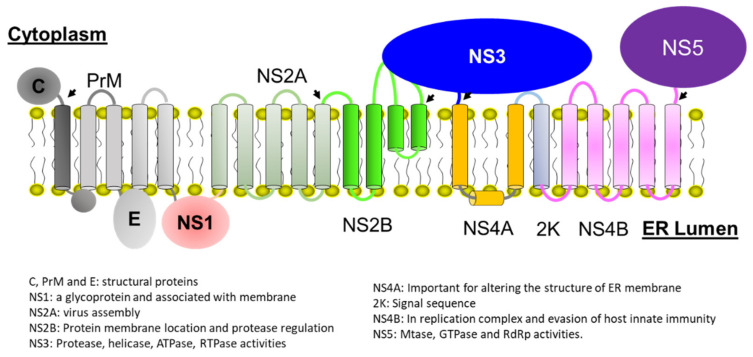
A schematic plot of Zika polyprotein on the membrane of the endoplasmic reticulum. Arrows indicate the viral protease cleavage sites. The cell membrane and membrane topology of viral proteins are listed. The possible transmembrane helices are indicated as cylinders. Different viral proteins are indicated in different color. The figure was made based on the previous report [33] and permission was obtained. For the detailed function of viral proteins, please refer to other references [18,30,37,39,68,69,70,71,72,73].

ZIKV is a positive-sense RNA virus whose genome has one open reading frame [12,13]. The polyprotein encoded by the viral genome is cleaved by both host and viral proteases to release functionally structural and non-structural proteins. ZIKV genome encodes three structural proteins, C, PrM/M, and E [14,15], and seven non-structural proteins, NS1, NS2A, NS2B, NS3, NS4A, NS4B, and NS5. The amino acid sequences of ZIKV proteins exhibit high sequence homology with those of other flaviviruses, such as dengue and West Nile viruses. The functions of viral proteins can be predicted based on the available knowledge of flaviviruses. Among these non-structural proteins, only NS3 and NS5 of flaviviruses possess enzymatic activities [16,17,18,19,20,21,22,23,24,25,26,27,28,29,30,31,32]. The other proteins are indispensable for viral invasion, assembly, and replication through interacting with cell membrane, host proteins, or viral protein–protein interactions [33,34,35,36,37,38,39,40,41,42,43,44,45,46] (Figure 1). The molecular interactions among these non-structural proteins are important for viral replication and sustaining the functions of some proteins, such as NS3 protease activity. Viral protease is responsible for cleaving the joints of NS2A/NS2B, NS2B/NS3, NS3/NS4A, and NS4B/NS5, which are at the cytoplasmic site of endoplasmic reticulum (ER) to release functionally non-structural proteins. Therefore, inhibiting the protease activity of ZIKV is a strategy to combat the virus [11,33,47,48,49]. As the sequence of ZIKV protease exhibits high homology/identity with those of dengue and West Nile viral proteases, ZIKV protease inhibitors might show the broad activity against these viral proteases [50,51,52,53]. Indeed, three-dimensional structures of these viral proteases are very similar, which further demonstrates that it is possible to develop protease inhibitors with a broad antiviral spectrum. ZIKV protease is a two-component protein complex formed by a membrane protein NS2B and an N-terminal portion of NS3 [54,55]. Structural studies have been carried out using different artificial constructs [56,57,58,59,60,61]. Similar to the proteases of dengue and West Nile viruses, conformational changes exist in ZIKV protease, while the closed conformation is predominant in the solution [62,63,64,65,66,67]. In this review, the functions and structures of the protease are summarized. We also summarize the development of protease inhibitors. The dynamics of residues in the protease is critical for its function and the conformational changes observed in structural studies provide useful information for rational design of small molecule inhibitors. 

## 2. Protease Structure and Dynamics

The protease of ZIKV is a serine protease with a catalytic triad formed by three residues, H51, D75, and S135, in the N-terminal region of NS3, whose C-terminal domain contains helicase and NTPase activities [33]. Unlike other serine proteases, which only contain a single polypeptide chain, the flavivirus protease, such as the ZIKV protease, is formed by NS2B—a membrane protein with four helices embedded in the cell membrane and the N-terminal region of NS3 containing the residues for peptide cleavage [48,74,75,76,77,78,79]. Studies show that the hydrolysis of the peptide bond requires the formation of NS2B–NS3 complex [67,80,81]. Although NS2B does not contain residues responsible for substrate cleavage and its transmembrane domains are not critical for the enzymatic activity, it has several roles indispensable to the functioning of viral protease. First, NS2B is critical for the folding of NS3 as NS3 alone is insoluble or unstructured when it is expressed in bacterial cells. NS2B binds tightly with NS3 through a hydrophilic region between the second and the third transmembrane helix [67,80,81]. Second, the molecular interaction between NS2B and the substrate is critical for the enzymatic activity, as recombinant protease constructs lacking the substrate-binding region from NS2B exhibited almost no protease activity. Lastly, NS2B is located on the cell membrane [82]. The NS2B–NS3 complex makes the protease approachable to other cleavage sites, which are in close proximity to cell membranes. The non-structural proteins of the virus form a replication complex on the ER membrane [83,84,85,86,87]. The location of NS2B on the membrane is critical for protease activity, which makes NS3 perform its other functions (important for viral replication). Therefore, both NS2B and NS3 are important targets for developing antivirals [88].

### 2.1. Structure of NS2B-NS3 Protease

**Figure 2:**
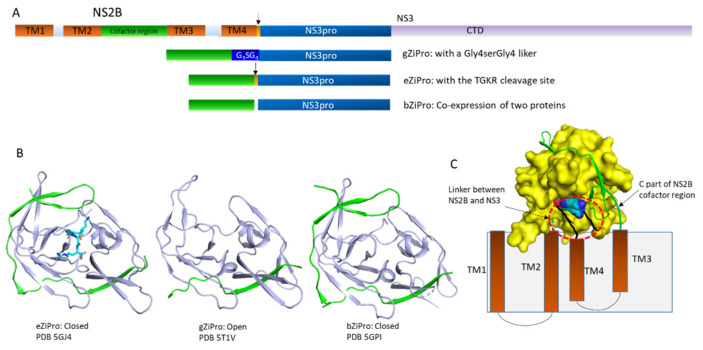
Open and closed conformations in ZIKV proteases. (**A**). Artificial constructs for structural studies of ZIKV protease. The domains of NS2B and NS3 are shown in different color. Transmembrane helices of NS2B are indicated as TM1-4. The cofactor region of NS2B interacting with NS3 and regulating protease activity is shown in green. The arrows indicate the native protease cleavage site between NS2B and NS3. The protease domain of NS3 is indicated as NS3pro and C-terminal region of NS3 is indicated as CTD. Artificial constructs without a linker, with a glycine-rich linker and the native protease cleavage site are indicated as bZiPro, gZiPro, and eZiPro, respectively. (**B**). Structures of the free ZIKV protease. Protease is in the closed conformation in the presence of an inhibitor. The NS2B cofactor region and NS3pro are shown in green and light blue, respectively. The PDB access codes are shown in the figure. All the structures are shown in the same orientation. The TGKR sequence is shown in sticks. (**C**). A model of ZIKV protease on the cell membrane. The transmembrane domains of NS2B are indicated as TMs. The boundary between NS2B and NS3 is highlighted with a red cycle. NS2B cofactor and NS3pro are shown in green and yellow, respectively. The dynamic nature of substrate is critical for its dissociation with protease, which is critical for releasing other proteins.

To explore the structure of viral proteases, different constructs were made for in vitro studies [89]. NS2B demonstrated to have four transmembrane helices while the folding of these helices is not defined in the structural study due to lack of long-range restraints [71,90]. The structure of the full-length NS2B fused with NS3 was not obtained, as the folding of the entire NS2B requires the presence of membrane systems and is challenging for crystallization. Studies of crystal structures on most flavivirus proteases used an artificial construct in which the hydrophilic NS2B peptide between transmembrane helices 2 and 3 was linked with the NS3 protease region (NS3pro) via a G_4_SG_4_ linker [91,92,93]. This construct was overexpressed and purified from bacterial cells for biochemical and structural analysis in the absence of membrane systems [80,81,94]. Structures of this construct reveal that protease exists in open and closed conformations under different conditions [67,95]. The folding of NS3 is almost identical in both conformations while the C-terminal portion of the NS2B region exhibits various conformations [87]. Structural studies show that proteases of dengue West Nile virus and ZIKV are very similar and adopt a chymotrypsin-like structure with two β-barrels. NS3 harbors the catalytic triad formed by three residues (H51, D74, and S135) [96]. Approximately 40 amino acids from NS2B form a complex with NS3pro. The N-terminal region of the NS2B peptide forms a β-strand and integrates with the N-terminal domain of NS3, and the binding affinity is very low, as NS2B and NS3pro always form a complex when they are co-expressed in bacterial cells [97,98]. It has been noted that the free 40-residue NS2B peptide was observed when the NS2B-NS3pro complex was purified for structural studies [57]. There is no cell-based study to show the presence of free NS2B. It might be useful for exploring the novel function of NS2B [99,100,101]. The C-terminal part of the 40-residue NS2B peptide forms a β-hairpin structure, wrapping around the active site of the protease to form the active-closed conformation in which NS2B participates in formation of the S2 pocket, which is crucial for substrate-binding and catalytic activity [57,58,59,60,61,102]. A structural study also reveals a salt bridge between the side chain of P2 residue and D83 of NS2B while such an interaction was not observed in structures of dengue and West Nile proteases [54]. In the absence of a substrate or an inhibitor, the C-terminal region of NS2B can stay away from the active site to form the inactive-open conformation in a construct containing the G_4_SG_4_ linker [56] (Figure 2). Further NMR studies demonstrate that both conformations are present in this construct and inhibitors/substrates are able to increase the population of the closed conformation in solution [62,63,65,66,103,104,105]. Another NMR study demonstrated that removal of the glycine-rich linker in the dengue protease can also increase the population of the closed conformation in solution [98]. In the study, the hydrophilic NS2B region, comprising approximately 40 residues, was co-expressed with NS3pro. The purified dengue protease exists mainly in the closed conformation in solution as evidenced by the paramagnetic study. The similar construct of ZIKV protease was studied and the result shows that the closed conformation is predominantly present while conformational exchanges exist for some residues at the interface of NS2B and NS3. Inhibitor or substrate binding to protease eliminates such exchanges [57,58,59,60]. The structural studies on the three structures provide useful information for designing inhibitors (Figure 2).

The structures of ZIKV protease have been well characterized using three constructs namely gZiPro with a G_4_SG_4_ linker, eZiPro with a native protease cleavage site containing P1 to P4 residues, and bZiPro without any linker between NS2B cofactor region and NS3pro [57,58,106]. Both bZiPro and eZiPro are in the closed conformation and the C-terminal region of the 40-residue NS2B peptide is well folded. In bZiPro, the active site is open for substrate binding. In eZiPro structure, P1–P4 residues occupy the active site and exhibit interactions with NS2B while the protease is still active and binds to inhibitors [57,58]. The open structure was observed in gZiPro and the inhibitor bound gZiPro exists in the closed conformation [54,107]. Several studies demonstrate that bZiPro is very suitable for understanding protease and inhibitor interactions, as the substrate-binding pocket is accessible to various types of inhibitors [57,59,60,102]. Although the active site of eZiPro is occupied by its native substrate, this construct is close to the native protease under physiological conditions. A study also shows that the hit rate of fragment screening using eZiPro is higher than that of bZiPro [102]. The artificial linker in gZiPro affects the chemical environment of quite a few residues, while it is still useful for structural and biochemical studies [61,108]. All constructs are very useful for developing protease inhibitors, while they are artificial constructs lacking the NS2B transmembrane regions, which can restrict the motion of the hydrophilic region [33,63,109,110]. 

The crystal structures of flaviviruses provide insight into drug design and a clue to evaluate whether an inhibitor can exhibit activity against other viral proteases in this family. The current ZIKV protease inhibitors also exhibit activity against proteases of dengue and West Nile viruses. To evaluate whether an inhibitor has a broad spectrum against different viruses in this family, analyzing protease sequences and structures will be a feasible strategy. 

### 2.2. Protease Druggability

**Figure 3:**
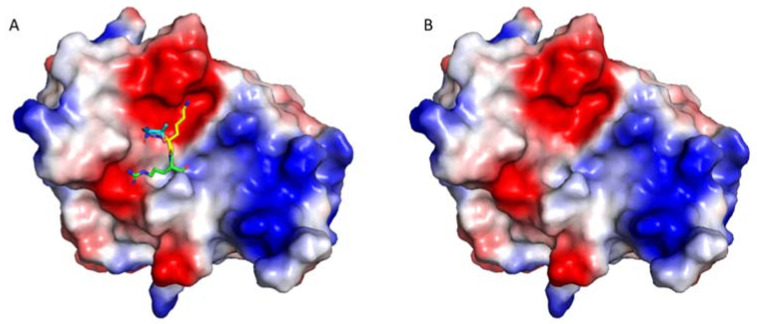
The protease active site is negatively charged. The structures of eZiPro (PDB ID 5GJ4) with (**A**) and without (**B**) TGKR sequence of NS2B are shown to understand the surface charges. The TGKR residues are shown in different color. The surface charge figure was made using PyMOL (www.pymol.org (accessed on 3 August 2018)). Surface areas with positive charge, negative charge, and no charge are shown in blue, red, and white, respectively. The substrate-binding site is negative charged, suggesting the challenges of developing small molecule inhibitors, which prefer interacting with a hydrophobic surface.

Druggability is a term in drug discovery referring to the likelihood of developing a small-molecule compound, which can modulate a target [111,112]. Druggability of a target can also be interpreted as the fact that a binding site is present for forming tight interactions with a small-molecule compound. It is important to estimate the successful rate of a drug discovery project. There are several ways to predict druggability of a target. The structural study on a target is one of the efficient strategies to analyze druggability [113,114]. A small-molecule drug is usually hydrophobic with a tendency to interact with a hydrophobic surface. Therefore, analyzing the surface charges of a target that affect the hydrophobicity of the pocket can be utilized as one of the criteria to evaluate the druggability [115]. Proteases of flaviviruses, such as ZIKV, dengue, and West Nile virus recognize a sequence with positively charges residues such as Arg and Lys at P1 and P2 position [116]. The S1 and S2 pockets of the protease active site are negatively charged (Figure 3). Therefore, the druggability of the active side of ZIKV protease is very low [117]. To develop small-molecule inhibitors, other druggable sites of ZIKV protease need to be identified [118,119]. Structural studies reveal the presence of the open and closed conformations, making it possible to develop small-molecule inhibitors through allosteric mechanisms, such as stabilizing the open/inactive conformation or inducing conformational changes of NS2B [74,120].

The charges in the protease active site make developing small molecules challenging while the progress made in the development potent peptidic and allosteric inhibitors still show that it is feasible to develop protease inhibitors for clinical applications.

### 2.3. Protease Dynamics

**Figure 4:**
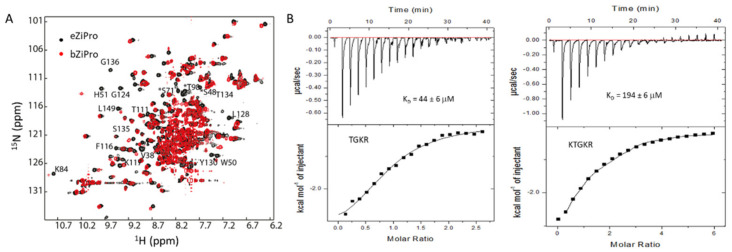
Dynamics and conformational changes in ZIKV protease. (**A**). Overlay of ^1^H-^15^N-HSQC spectra of eZiPro and bZiPro. This figure is obtained from the reference [58]. The ^1^H-^15^N-HSQC spectra of eZiPro and bZiPro are shown in black and red, respectively. More cross-peaks appeared in eZiPro suggests that substrate binding to protease suppresses exchanges. (**B**). Binding affinity between protease and peptides. The weak binding affinity is important for the function of the protease. This figure was obtained from the reference [108] with permission.

The native form of ZIKV protease contains four components—the transmembrane helices of NS2B, the 40-residues NS2B sequence (cofactor region) interacting with NS3pro, the protease cleavage site, and NS3pro (Figure 2). The structural study has not yet been carried out for the native form of ZIKV protease [110,121,122]. Protein dynamics and conformational changes have been analyzed using artificial constructs and computational techniques [64,123,124]. The first NMR study on West Nile protease was carried out to understand the open and closed conformations [62]. In the study, exchanges were observed in the protease and inhibitor binding to protease stabilized the closed conformation. As the protease cleavage site is present in the native protease, the closed conformation was proposed to be utilized in structure-based drug design [65,66]. A careful study was carried out on dengue protease to access the folding of the protease in solution. Both open and closed conformations were observed in solution [103]. Similar to West Nile protease, inhibitor binding to dengue protease reduces the population of the closed conformation [63]. Another NMR study using an unlinked dengue protease demonstrated that the closed conformation was predominant in solution [98] (Figure 4). In the unlinked protease-bZiPro, the protease exists in the closed conformation while exchanges are also present for the residues at the C-terminal part of NS2B peptide and some residues from NS3 (Figure 4). In the presence of substrate peptides, the exchanges at the NS2B and NS3pro were suppressed (Figure 4). A crystal structure of eZiPro captured the molecular interactions between P1–P4 residues and viral protease [58]. A follow-up NMR study demonstrates that the P1–P4 residues are flexible in eZiPro [108]. Indeed, binding studies using an unlinked protease-bZiPro show that peptides derived from the protease cleavage site (P1–P4 residues) bind to protease with affinities in µM–mM range [108] (Figure 4). Such a dynamic nature and weak protease binding affinity of the residues at the cleavage site is critical for the protease’s function as these residues are present in the native protease and should not interfere with the cleavage of other positions (Figure 1). The dynamic nature might be important for viral protease to recognize cleavage sites. Inhibitor/substrate induced conformational changes were observed in proteases of dengue, West Nile, and Zika viruses. In addition, computation-based studies also reveal the structural changes of viral proteases [125,126]. Based on these observations, researchers have been developing compounds that were able to stabilize the inactive/open conformation [49,127]. As the protease contains two proteins, any compounds affecting their interactions might be active against the enzymatic activity. The dynamics and conformational changes of the protease revealed by structural studies provide insights into developing protease inhibitors [128].

In summary, the dynamics of proteases is critical for function of the enzymes and provides insights into inhibitor design. First, the open and closed conformations observed in the crystal structures provide ways to develop inhibitors. Any compounds able to lock these conformations will be effective in inhibiting the enzyme activity. Second, the weak binding affinity between NS3 and P1–P4 residues, which are dynamic in solution, is critical for the enzyme to cleave other enzymatic cleave sites. Lastly, the dynamic nature of the protease might be critical for changing orientations of the protease on the membrane, which can be critical for the protease activity or other enzymatic activities of NS3.

## 3. Protease Inhibitors

**Figure 5:**
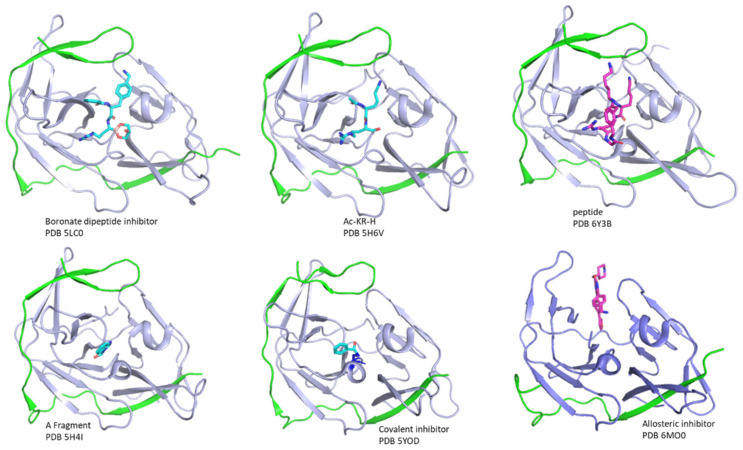
Structures of several protease-inhibitor complexes. The structures of inhibitors in complexes with proteases are shown. NS2B and NS3 are shown in green and light blue, respectively. The PDB accessing codes are indicated in the figure. All the structures are shown in the same orientation. Several types of inhibitors such as peptidic inhibitors, fragments, irreversible inhibitors, and allosteric inhibitors are shown in sticks.

Several strategies, such as high throughput screening (HTS), computation-guided drug design, and fragment based drug discovery have been applied to develop protease inhibitors [129,130,131,132,133]. The available inhibitors have been reviewed thoroughly [10,33,117,134,135,136,137,138,139]. Inhibitors include substrate derived peptidic compounds, small molecules binding to the active site and allosteric inhibitors (Figure 5) [11]. Due to the presence of conformational exchanges in the protease and low druggability of the protease active site [89], developing the allosteric inhibitor is of great interest and potent allosteric inhibitors have been developed [50,118,140,141]. As this type of inhibitor exhibited antiviral activity in cell-based assays, it has great potential in being developed into antivirals.

### 3.1. Peptidic Inhibitors

Inhibitors derived from the substrate have been developed and well-characterized [142,143,144]. These peptidic inhibitors can grouped into three classes: covalent peptidic inhibitors, cyclic peptides, and normal peptides. Covalent peptidic inhibitors are the dominant ones and contain three important regions—the backbone derived from the substrate sequence interacting with the S1–S2/S3 sites, the cap region enhancing the activity by forming interactions with residues outside of S1 and S2/S3 sites, and a warhead at the C-terminus, such as aldehyde and boronic acid forming a covalent bond with residue S135 [93,145,146,147]. One of the most characterized peptidic inhibitors is a tetrapeptide inhibitors-nKRR-aldehyde, which exhibited a wide-spectrum activity against flavivirus proteases [59,60,116]. Extensive studies, on, e.g., optimizing the competent of the peptide sequence using different amino acids, adopting various warheads, and shortening or increasing the length of the peptide, have been carried out to improve the potency of inhibitors [148,149]. The peptidic inhibitor with the smallest molecule weight is Ac-KR-aldehyde in which two amino acids are present [60]. Inhibitors that are cyclic peptides can be developed from the substrates and identified from screening. This type of inhibitor usually contains more than three amino acids. The cyclic peptides can be more stable than normal peptides under physiological conditions. It is possible to develop both competitive and noncompetitive cyclic peptides, while extensive studies are needed to improve the potency [57,119]. Developing linear peptides active against the ZIKV protease will be challenging, while a small protein bovine pancreatic trypsin inhibitor (BPTI) is active against proteases from several viruses [58]. Despite efforts being made in drug discovery, there are still no peptidic inhibitors suitable for further studies, which is due to the fact that the charged residues at the P1 and P2 positions cannot be replaced [116]. Therefore, the peptidic inhibitor exhibited potent inhibitory activity against ZIKV protease while challenges in stability in vivo and penetrating the cell membrane make them difficult for clinical studies. Peptides that can be utilized in clinical studies need to be developed [10,119].

Although it is challenging to develop peptidic inhibitors, quite a few completive and noncompetitive peptidic inhibitors are available. The following strategies might be useful to develop antivirals derived from peptides. First, adoption of unnatural amino acids in inhibitors. Second, exploring cyclic peptidic inhibitors is a feasible strategy to improve the properties of the inhibitors. Third, linking peptidic inhibitors with small molecules might be a promising strategy while extensive structural studies are needed. Lastly, allosteric peptidic inhibitors are of great interest while structural studies are important for elucidating the mechanism of action.

### 3.2. Small-Molecule Inhibitors

Strategies, such as virtual screening and HTS campaigns, have been applied to identify small molecule inhibitors [129,150]. Although quite a few inhibitors are available [150,151], no compound has reached clinical studies. Due to the hydrophilic nature of the protease, these small-molecule inhibitors that bind to the active site are not as potent as peptidic inhibitors, unless they form a covalent bond with the protease. The following types of small molecules are developed. First, some small-molecule inhibitors are developed using conventional methods, such as HTS and structure-based design. Quite a few fragments were identified and shown to bind to the substrate-binding site while further fragment optimization is needed to improve the potency [57,102]. Second, developing irreversible inhibitors is a strategy to identify competitive inhibitors. An irreversible inhibitor was shown to be effective in inhibiting the Zika protease by forming covalent interactions with Ser135 [59,152]. Structural studies and mass spectrometry analysis revealed that a portion of the compound formed a covalent bond with S135. Despite its potency against protease activity, this compound is still difficult for clinical studies due to its stability. Third, allosteric inhibitors can be developed targeting ZIKV protease. Several inhibitors were reported to inhibit protease inhibitors through allosteric or noncompetitive manners. These inhibitors were predicted to interact with a druggable site [50,118,153]. Recent reports show that a series of 2,5.6-trisubsitituted pyrazine compounds are potent Zika protease inhibitors by regulating the enzymatic activity through an allosteric mechanism. The nanomolar IC_50_ observed in the biochemical assay and low micromolar EC68 observed in the cell-based assay suggested that these compounds have great potency to be applied in clinical studies [50]. The available crystal structure of an allosteric inhibitor bound to dengue protease proves its mode of action [50,52]. An assay was developed to screen allosteric inhibitors. This assay was based on the presence of the open and closed conformations in ZIKV protease. As the C-terminal region of NS2B exhibits conformational changes, a conformational switch assay was developed. Several allosteric inhibitors were screened using this assay. An identified inhibitor-NSC135618 was shown to be an allosteric inhibitor with a broad spectrum [154]. The allosteric inhibitors have great potential to be developed into antivirals as they exhibited activity in cell-based assays. Structural studies and biophysical studies are still required to understanding their binding modes. Lastly, drug repurposing was applied to identify ZIKV protease inhibitors [52]. Several drugs were shown to be active against the ZIKV protease, while it is challenging to further optimize these drugs without structural information.

Protease inhibitors can be developed using strategies, such as HTS, fragment-based drug design, drug repurposing, and structure-based drug design. Due to the hydrophilic nature of the substrate-binding site, designing allosteric inhibitors is a feasible strategy to develop antivirals against ZIKV infection. It important to have an assay available to identify noncompetitive/allosteric molecules. Structures and dynamics of the protease in complexes with compounds will be critical for optimization. With novel strategies utilized in compound screening and compound optimization, more potent Zika protease inhibitors will be obtained [104,133,141,155,156,157].

## 4. Strategies in Inhibitor Design

Several ZIKV protease inhibitors have been designed while no compound has been reached into clinical studies. Different types of screening strategies including HTS using biochemical and cell-based assays, fragment-based screening, drug repurposing and virtual screening and structure-guided drug design have been utilized to identify potent protease inhibitors [10,11,158,159,160]. Nonetheless, some compounds active in biochemical assays did not exhibit any activity in cell-based assays [161]. Although this can be attributed to the chemical properties of the compounds, suitable assays in evaluating protease activity and probing protein–ligand interactions will be important in this field. There are three artificial constructs available for ZIKV protease [33], but the native form of ZIKV protease might be needed for evaluating the activity of the inhibitor and the screening of a compound from the compound library. An assay that can measure the protease and ligand binding in living cells is also helpful for evaluating and identifying new inhibitors [154].

The protease contains some regions with exchanges, which are important for the protease function and for designing potent compounds [154,162]. The P1–P4 residues at the native protease cleavage site at the joint of NS2B and NS3 is dynamic in solution, giving rise to empty the protease pocket for interacting with other sites. Therefore, developing a compound that can stabilize the active form with the active site occupied is a good strategy to develop inhibitors [10,11,52]. As aforementioned, this strategy is challenging due to the low druggability of the protease active site. The dynamic feature of the protease makes it possible to develop allosteric inhibitors, which are able to stabilize the inactive conformation and destabilize the active conformation. A recent study demonstrated the feasibility of this strategy [50,52,118,153]. With the availability of structural information by X-ray crystallography, dynamic analysis by solution NMR spectroscopy, computational analysis, biochemical assays, and cell-based assays, developing allosteric inhibitors is an effective and promising strategy in antiviral development.

## 5. Conclusions

Quite a few viral proteins, such as ZIKV protease, possess regions that are dynamic in nature, which is critical for their functioning. Such dynamic information provides a clue for rational inhibitor design. To obtain accurate structural and dynamic information, the following factors need to be considered: a suitable construct for in vitro studies, an appropriate assay for measuring activity, a sensitive analytical tool to understand dynamics, and a cell-based assay to validate the observations. With the accumulated structural information of viral proteins and developed computational methods, the dynamics of viral proteins will be considered in rational drug design. More allosteric inhibitors can be developed by rational design.
